# The Validity of Generative Artificial Intelligence in Evaluating Medical Students in Objective Structured Clinical Examination: Experimental Study

**DOI:** 10.2196/79465

**Published:** 2025-12-04

**Authors:** Masashi Yokose, Takanobu Hirosawa, Tetsu Sakamoto, Ren Kawamura, Yudai Suzuki, Yukinori Harada, Taro Shimizu

**Affiliations:** 1 Department of Diagnostic and Generalist Medicine Dokkyo Medical University Tochigi Japan; 2 Department of Internal Medicine Yamagata Prefectural Kahoku Hospital Yamagata Japan

**Keywords:** objective structured clinical examination, medical education, generative artificial intelligence, ChatGPT-4, OSCE, medical students

## Abstract

**Background:**

The Objective Structured Clinical Examination (OSCE) has been widely used to evaluate students in medical education. However, it is resource-intensive, presenting challenges in implementation. We hypothesized that generative artificial intelligence (AI) such as ChatGPT-4 could serve as a complementary assessor and alleviate the burden of physicians in evaluating OSCE.

**Objective:**

By comparing the evaluation scores between generative AI and physicians, this study aims to evaluate the validity of generative AI as a complementary assessor for OSCE.

**Methods:**

This experimental study was conducted at a medical university in Japan. We recruited 11 fifth-year medical students during the general internal medicine clerkship from April 2023 to December 2023. Participants conducted a mock medical interview with a patient experiencing abdominal pain and wrote patient notes. Four physicians independently evaluated the participants by reviewing medical interview videos and patient notes, while ChatGPT-4 was provided with interview transcripts and notes. Evaluations were conducted using the 6-domain rubric (patient care and communication, history taking, physical examination, patient notes, clinical reasoning, and management). Each domain was scored using a 6-point Likert scale, ranging from 1 (very poor) to 6 (excellent). Median scores were compared using the Wilcoxon signed-rank test, and the agreement between ChatGPT-4 and physicians was assessed using intraclass correlation coefficients (ICCs). All *P* values <.05 were considered statistically significant.

**Results:**

Although ChatGPT-4 assigned higher scores than physicians in terms of physical examination (median 4.0, IQR 4.0-5.0 vs median 4.0, IQR 3.0-4.0; *P*=.02), patient notes (median 6.0, IQR 5.0-6.0 vs median 4.0, IQR 4.0-4.0; *P*=.002), clinical reasoning (median 5.0, IQR 5.0-5.0 vs median 4.0, IQR 3.0-4.0; *P*<.001), and management (median 6.0, IQR 5.0-6.0 vs median 4.0, IQR 2.5-4.5; *P*=.002), there were no significant differences in the scores of patient care and communication (median 5.0, IQR 5.0-5.0 vs median 5.0, IQR 4.0-5.0; *P*=.06) and history taking (median 5.0, IQR 4.0-5.0 vs median 5.0, IQR 4.0-5.0; *P*>.99), respectively. ICC values were low in all domains, except for history taking, where the agreement was still poor (ICC=0.36, 95% CI –0.32 to 0.78).

**Conclusions:**

ChatGPT-4 produced higher evaluation scores than physicians in several OSCE domains, though the agreement between them was poor. Although these preliminary results suggest that generative AI may be able to support assessment in some domains of OSCE, further research is needed to establish its reproducibility and validity. Generative AI like ChatGPT-4 shows potential as a complementary assessor for OSCE.

**Trial Registration:**

University Hospital Medical Information Network Clinical Trials Registry UMIN000050489; https://center6.umin.ac.jp/cgi-open-bin/ctr/ctr_his_list.cgi?recptno=R000057513

## Introduction

Patient encounters, including history taking and physical examination, are fundamental skills in clinical medicine. High-quality patient encounters can yield numerous benefits, including timely and accurate diagnoses, a comprehensive understanding of a patient’s status, the establishment of rapport between physicians and patients, and the development of optimal treatment strategies [1]. Given their importance, evaluating and ensuring the competency of health professionals in conducting these encounters is essential.

The Objective Structured Clinical Examination (OSCE) is one of the most widely accepted, objective, and standardized assessment tools in medical education [2]. It has been extensively employed to assess clinical competencies such as communication, physical examination, clinical reasoning, and procedural techniques [3]. OSCE typically consists of a series of time-limited stations where candidates perform specific clinical tasks under observation, often involving interactions with standardized patients (SPs). By simulating real-life clinical situations in a controlled environment, OSCE enables consistent assessment and provides the opportunity to evaluate a wide range of clinical skills [3]. As such, it contributes to improving the validity and reliability of clinical skills assessment, supporting the application of theoretical knowledge in practical, patient-centered care [4].

Despite its benefits, OSCE has some disadvantages. A major challenge is its high demand for human and logistical resources. Effective implementation requires trained examiners, SPs, administrative staff, and physical infrastructure [3,5,6]. Preparing SPs and ensuring objective scoring are both time- and labor-intensive. Financial costs are also substantial, with reports estimating up to €10,000 (about US $11,598) per exam and over €100 (about US $115) per examinee [7]. Furthermore, despite standardization efforts, interrater variability and scoring subjectivity can undermine assessment reliability [3,8]. These challenges have led to implementation difficulties in some settings [9].

In recent years, generative artificial intelligence (AI), particularly large language models, has shown potential in medicine [10]. In the context of medical education, such models may offer benefits including personalized feedback, enhanced simulation, and support for self-directed learning [11]. One emerging application is the use of AI to assist with or complement assessment processes in OSCEs. Although AI cannot currently replicate the full range of human evaluators’ observational abilities, it may help reduce certain aspects of examiner workload such as providing structured feedback or scoring text-based materials [12]. However, the validity of AI-based assessment remains underexplored, particularly when compared with experienced human judgment.

This study aims to provide preliminary evidence of the validity of generative AI in evaluating the performance of medical students in OSCE. By comparing the evaluations between physicians and AI, we aim to explore the feasibility and potential limitations of generative AI as a supplemental tool for OSCE evaluation.

## Methods

### Overview

We conducted an experimental study to assess ChatGPT-4's evaluation of students' mock medical interviews compared to the evaluation by physicians. This research was conducted at the Department of Diagnostic and Generalist Medicine (General Internal Medicine), Dokkyo Medical University, Tochigi, Japan.

### Ethical Considerations

This study followed the Declaration of Helsinki. The study protocol was approved by the institutional review board of Dokkyo Medical University (approval 2022-015). All participants provided written informed consent prior to participation. They were informed that their participation was voluntary and that they could withdraw from the study at any time without penalty. Participants received a 2,000-yen (about US $12.80) coffee ticket as compensation. All components, including the medical interviews, their transcriptions, the structured prompts, and outputs from ChatGPT-4, were in Japanese. To safeguard privacy, all data were anonymized before use. To prevent biases from previous interactions, we deactivated the settings related to ChatGPT-4's chat history and training controls. Moreover, each session was restarted before conducting a new evaluation. This approach ensured a single, unbiased output from ChatGPT-4 for each medical interview. Our research methods included recruiting participants, conducting mock medical interviews, transcribing and verifying the data, inputting the data into ChatGPT-4, and evaluation by physicians.

### Participant Recruitment

We recruited fifth-year medical students who had rotated through the General Internal Medicine department at Dokkyo Medical University. Unlike the 4-year medical curriculum in many countries, Japan follows a 6-year medical curriculum. Typically, Japanese medical students learn basic medicine in the first two years and clinical medicine in the following two years. After passing the standard achievement tests in Japanese and OSCE, they attend clinical clerkships in the last two years. Written informed consent was obtained from each medical student a week after the study's protocols were explained.

### Conducting Mock Medical Interviews

Participants took a face-to-face mock medical interview with a patient experiencing intermittent abdominal pain in the primary care setting. The scenario was originally developed based on a textbook of the United States Medical Licensing Examination Step 2 Clinical Skills [13] and had been implemented in our general internal medicine clerkship for 2 years prior to the study with regular updates. At the beginning of the interview, participants had 1 minute to review the patient’s basic information, including age, sex, chief complaints, and vital signs. Then, participants underwent a 12-minute history taking and physical examination. After the interview, participants completed patient notes on the laptop in 15 minutes. We prepared a template for the patient note, and participants filled in patient history, physical examination findings, assessments (including up to 3 differential diagnoses with supporting evidence), and management plan. We allocated the time based on the United States Medical Licensing Examination Step 2 Clinical Skills [13] and the OSCE that sixth-year medical students take after clinical clerkships in Japan [14]. We recorded each interview. Interviews were conducted in Japanese, and participants completed patient notes in Japanese.

### Transcribing and Verifying the Data

The leading researcher (MY) reviewed medical interview videos and transcribed the conversation verbatim. Another researcher (TH) then reviewed the same interview videos to verify the accuracy of the transcripts and to finalize them. To clarify who was speaking in the conversation, we added the headers “Physician” or “Patient” to each conversation in the medical interview. The names of the medical students, as they introduced themselves in the medical interview, were changed to pseudonyms for privacy protection. Any discrepancies such as transcription differences between MY and TH were discussed and resolved. We present examples of the transcripts (Textbox 1) and patient notes (Textbox 2).

**An example of the transcripts of the medical interview. This is a part of the English translation of the original transcripts written in Japanese.**

…

Physician: Thank you. So, what brings you in today?

Patient: Oh, today, I came because I have a bit of a stomachache.

Physician: A stomachache. I see. Could you tell me a little more about this stomach pain?

Patient: Please ask me anything, Doctor.

Physician: Okay, then. I assume there was a point when the pain started?

…

**An example of patient notes. This is a part of the English translation of the original patient notes written in Japanese.**

(History)

Epigastric pain has been present for the past two weeks. The pain has been worsening and continues to persist.

…

(Physical examination findings)

Consciousness: Clear and coherent.

General appearance: Tends to be obese.

…

(Assessment)

Differential diagnosis #1: gastric ulcer

Supporting history: Epigastric pain that worsens after meals, particularly with pizza and hamburger intake

…

(Plan)

Blood tests (including C-Reactive Protein, WBC)

Upper gastrointestinal endoscopy

### Inputting Data Into ChatGPT-4

We selected ChatGPT-4 with ChatGPT Plus plan for this study due to its advanced natural language processing capabilities and its potential in various fields, including health care. ChatGPT-4, developed by OpenAI, is recognized for its ability to understand and generate human-like text, making it a suitable tool for evaluating the complexities involved in medical interviews. Its multilingual support, including Japanese, further enabled us to conduct this research effectively within our local context [15]. We accessed ChatGPT-4 by using an individual ChatGPT Plus account. The access period was January 11, 2024. We input the transcribed medical interviews along with patient notes and structured prompts into ChatGPT-4. Patient notes included assessment and plan for the mock case. We created structured prompts based on a preliminary investigation, and each prompt combined the interview transcript with specific evaluation criteria. We adopted an evaluation format derived from the OSCE in Japan [16], which consisted of the following 6 items: patient care and communication, history taking, physical examination, patient notes, clinical reasoning, and management. For each aspect, ChatGPT-4 was asked to evaluate based on a scale of 1 to 6, with 1 being the lowest and 6 being the highest. The criteria for each score were clearly defined. To prevent biases from previous interactions, we deactivated the settings related to ChatGPT-4's chat history and training controls. Moreover, each session was restarted before conducting a new evaluation. This approach ensured a single, unbiased output from ChatGPT-4 for each medical interview. Details of the prompt are provided in Multimedia Appendix 1.

### Evaluation by Physicians

We used a consensus-based approach in which 2 pairs of physicians (T Sakamoto and YS, RK and YH) were randomly allocated to the evaluation of 5 or 6 participants. Then, each physician independently reviewed medical interview videos and patient notes and evaluated the participants based on the same criteria as ChatGPT-4. Any disagreements within the pair regarding the evaluation were resolved through discussion.

### Data Collection and Outcome

We collected data on participants’ sex and prior experience with medical interviews. The primary outcome was the difference in the evaluation scores between ChatGPT-4 and physicians in each domain.

### Statistical Analysis

The evaluation scores by physicians and ChatGPT-4 were presented as median with 25th and 75th percentiles and were compared using the Wilcoxon signed-rank test. We calculated intraclass correlation coefficients (ICCs) and their 95% CIs to evaluate the agreement between ChatGPT-4 and physician evaluations (ie, interrater reliability). A 2-way random-effects model with absolute agreement was used, based on a single measurement across 11 participants. Generally, the degree of reliability is estimated along with the following ICC values: <0.5=poor; 0.5-0.75=moderate; 0.75-0.9=good; >0.9=excellent [17]. All *P* values <.05 were considered statistically significant. We used R software (version 4.3.1; The R Foundation for Statistical Computing) for statistical analysis.

## Results

### Baseline Characteristics

**Figure 1:**
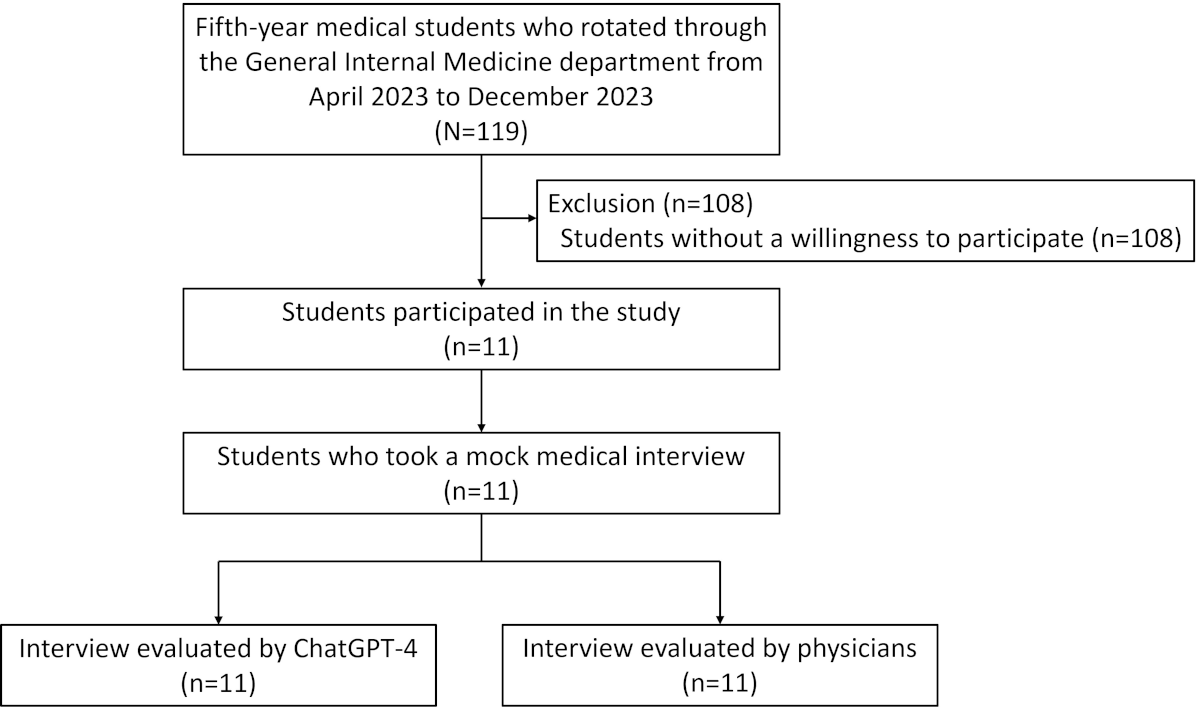
Study flow diagram of the experimental evaluation of generative artificial intelligence in medical education. The study was conducted at Dokkyo Medical University, Japan, between April and December 2023. A total of 11 fifth-year medical students were recruited during their general internal medicine clerkship. Each participant completed a mock medical interview with a standardized patient. Physicians evaluated each student by using both the video recordings and written patient notes, while ChatGPT-4 conducted its evaluation based on interview transcripts and patient notes.

From April 2023 to December 2023, 119 fifth-year medical students rotated through the General Internal Medicine department at Dokkyo Medical University, and 11 students participated in this study (Figure 1). All participants (11/11) had experienced medical interviews less than 10 times before this study, and 55% (6/11) of the participants were females.

### Evaluation Outcomes

Table 1 summarizes the comparison of the evaluation scores between ChatGPT-4 and physicians. With the 6-item evaluation format, ChatGPT-4 assigned higher scores than physicians in terms of physical examination (median 4.0, IQR 4.0-5.0 vs median 4.0, IQR 3.0-4.0; *P*=.02), patient notes (median 6.0, IQR 5.0-6.0 vs median 4.0, IQR 4.0-4.0; *P*=.002), clinical reasoning (median 5.0, IQR 5.0-5.0 vs median 4.0, IQR 3.0-4.0; *P*<.001), and management (median 6.0, IQR 5.0-6.0 vs median 4.0, IQR 2.5-4.5; *P*=.002). There were no significant differences in the scores for patient care and communication (median 5.0, IQR 5.0-5.0 vs median 5.0, IQR 4.0-5.0; *P*=.06) and history taking (median 5.0, IQR 4.0-5.0 vs median 5.0, IQR 4.0-5.0; *P*>.99), respectively. The ICCs and their 95% CIs were –4.27e-16 (–0.281 to 0.449) in patient care and communication, 0.359 (–0.318 to 0.780) in history taking, –0.029 (–0.258 to 0.386) in physical examination, –0.093 (–0.220 to 0.275) in patient notes, 0.033 (–0.038 to 0.232) in clinical reasoning, 0.120 (–0.105 to 0.502) in management, and 0.000 (–0.072 to 0.203) in the total score.

**Table 1:** Comparison of the evaluation scores between physicians and ChatGPT-4 across 6 domains in the Objective Structured Clinical Examination. Scores were assigned on a 6-point Likert scale, ranging from 1 (very poor) to 6 (excellent), for each domain. Total scores were also calculated. All *P* values <.05 were considered statistically significant.

	ChatGPT-4, median (IQR)	Physicians, median (IQR)	*P* value^a^	ICC^b^ (95% CI)
Patient care and communication	5.0 (5.0-5.0)	5.0 (4.0-5.0)	.06	–4.27e-16 (–0.281 to 0.449)
History taking	5.0 (4.0-5.0)	5.0 (4.0-5.0)	>.99	0.359 (–0.318 to 0.780)
Physical examination	4.0 (4.0-5.0)	4.0 (3.0-4.0)	.02	–0.029 (–0.258 to 0.386)
Patient notes	6.0 (5.0-6.0)	4.0 (4.0-4.0)	.002	–0.093 (–0.220 to 0.275)
Clinical reasoning	5.0 (5.0-5.0)	4.0 (3.0-4.0)	<.001	0.033 (–0.038 to 0.232)
Management	6.0 (5.0-6.0)	4.0 (2.5-4.5)	.002	0.120 (–0.105 to 0.502)
Column total	30 (29-31)	24 (20-26)	<.001	0.000 (–0.072 to 0.203)

^a^Statistical comparisons were conducted using the Wilcoxon signed-rank test.

^b^ICC: intraclass correlation coefficient.

## Discussion

### Principal Results

This experimental study compares evaluation scores between ChatGPT-4 and physicians in OSCE by using 6-item evaluation criteria. ChatGPT-4 assigned higher scores in the domains of physical examination, patient notes, clinical reasoning, and management, while no significant differences were found for history taking and patient care and communication. The low ICCs indicated poor agreement between ChatGPT-4 and physician evaluations in most domains. These results suggest that although ChatGPT-4 can generate structured assessments, its agreement with human raters is limited.

The higher ratings given by ChatGPT-4, particularly for physical examination skills, may be attributed to differences in the input material. Physicians evaluated student performance by using both video recordings and patient notes, allowing them to assess the visual and behavioral aspects of physical examination. In contrast, ChatGPT-4 evaluated students solely on interview transcripts and written notes. The absence of visual data likely contributed to “coarse-grained” or inflated evaluations by ChatGPT-4. Similarly, ChatGPT-4 also gave high ratings to the domains of patient notes, clinical reasoning, and management. As shown in Multimedia Appendix 1, structured prompts led ChatGPT-4 to assess these skills based solely on information from patient notes. In contrast, physicians may have evaluated deeper clinical consistency across sources, which could have contributed to stricter scoring. These discrepancies highlight the importance of input parity in comparative assessment studies.

We evaluated the agreement between ChatGPT-4 and physician evaluations by using ICCs as a measure of interrater reliability. The point estimates of ICCs were extremely low across most domains except for history taking (0.359, 95% CI –0.318 to 0.780). These findings indicate poor agreement overall and raise concerns about the construct validity of AI-based scoring in this context. The observed discrepancies might be attributed to some reasons, including the small sample size, differences in input modality between ChatGPT-4 and physicians, and the inherent limitations of AI in evaluating nonverbal or visual cues. Despite such limitations, the slightly higher ICC in history taking suggests that generative AI might show promise in evaluating domains that rely primarily on text-based information. Nonetheless, our results highlight the need for further validation before generative AI can be considered as a supplemental assessor for OSCE.

While preliminary, this study implies that generative AI may be cautiously explored as a complementary tool in OSCE scoring. Evaluations by AI could provide an additional perspective or be used as a screening before evaluations by physicians. Supported by generative AI, we could develop hybrid evaluation systems where generative AI and human evaluators collaborate to enhance the overall quality and reliability of the assessments.

### Limitations

This study has several limitations. First, it was conducted in Japanese, which may limit the generalizability of the findings to settings involving other languages. Second, the sample size of medical students was small, which restricts the statistical power and internal validity of the results. For example, the absence of significant score differences in history taking and patient care and communication may be attributable to insufficient power. Third, we used a single OSCE scenario involving abdominal pain, which limits generalizability across other clinical contexts. Although this scenario had not undergone external validation, it had been used in our department, suggesting a degree of internal content validity. Fourth, participation was voluntary, raising the possibility of selection bias. Fifth, we used ChatGPT-4 with ChatGPT Plus plan, and the results may not extend to other generative AI models or account types with different data privacy guarantees. Sixth, ChatGPT-4 and physicians had access to different input materials: while physicians evaluated both medical interview videos and patient notes, ChatGPT-4 evaluated only transcripts and notes. At the time of data collection (January 11, 2024), ChatGPT-4 did not support video input, necessitating the use of text-based inputs. This discrepancy likely influenced evaluations, particularly in domains such as physical examination and communication, which rely heavily on visual and nonverbal cues. To improve future study designs, it is essential to standardize input materials across evaluators. One possible approach would be a 3-arm comparison: (1) AI evaluating transcripts, (2) humans evaluating transcripts, and (3) humans evaluating videos. Although human-video evaluation is commonly treated as the gold standard in OSCE contexts, it too has limitations such as difficulty capturing subtle verbal or behavioral nuances. Acknowledging these constraints encourages a more cautious interpretation of comparative findings. Seventh, certain competencies evaluated in OSCE, particularly those that require visual or nonverbal confirmation (eg, physical examination techniques or nonverbal communication skills), are inherently unevaluable by text-only AI models. In our study, ChatGPT-4 evaluated all domains by using only transcripts and patient notes. Therefore, it was structurally incapable of detecting inconsistencies between documented notes and observed behaviors. This introduces a critical limitation for AI-based scoring of multimodal clinical skills. To address this issue, future studies should consider excluding domains that require multimodal inputs from AI-based assessments. Ensuring alignment between competency type (eg, verbal vs nonverbal) and AI capability will be critical to the appropriate use of generative AI in medical assessment. Eighth, we did not assess the reproducibility of ChatGPT-4’s evaluations. The specific version of ChatGPT-4 used is no longer available via the same interface, preventing retesting under consistent conditions. As a result, the stability of the AI assessments remains unclear. Ninth, physician ratings were determined through a consensus approach, which prevents evaluation of their reliability.

### Comparison With Prior Work

While there has been little evidence investigating the possibility of generative AI as an assessor in OSCE, some literature has evaluated the role of generative AI in simulated patient encounters. One report from the United States [18] demonstrated the implementation of ChatGPT-3.5 in training for breaking bad news, in which ChatGPT-3.5 created a scenario, performed as an SP, and gave immediate feedback to the trainee along with the SPIKES model. Another report by Misra and Suresh [12] demonstrated that ChatGPT generated feedback to the trainee based on the checklist completed by SPs in the OSCE scenario. In our study, we evaluated the potential of generative AI as a complementary assessor of OSCE by comparing assessments made by generative AI with those made by humans. The results suggest that generative AI can complement traditional evaluation methods and address some of the limitations associated with human assessments.

### Future Direction

Future research should expand both the sample size and the diversity of medical students and OSCE scenarios to enhance the generalizability of the findings. To determine whether observed score differences are attributable to evaluator type rather than input modality, it is essential to standardize input materials between human and AI evaluators. Moreover, aligning the types of competencies assessed in OSCE (eg, verbal vs nonverbal) with the capabilities of generative AI is critical for valid evaluation. The reproducibility of generative AI assessments should also be examined by performing repeated runs with the same inputs. These analyses will help clarify the stability and limitations of the evaluations by generative AI. In addition, future studies should explore hybrid assessment models that integrate AI with human evaluators, potentially combining the efficiency of automation with the nuance of expert judgment. Generative AI may also be valuable as a self-assessment tool for medical students during self-directed learning. Although generative AI shows promise in supporting medical student evaluation, it is crucial to recognize its limitations and ensure that it complements, rather than replaces, the essential role of human judgment in medical education.

### Conclusions

This experimental study shows that ChatGPT-4 assigned significantly higher ratings than physicians in 4 OSCE domains: physical examination, patient notes, clinical reasoning, and management. No significant differences were observed in the domains of history taking and patient care and communication. Although the results suggest that ChatGPT-4 can generate structured assessments, the low ICC values indicate that its agreement with physicians is limited. Further research with larger, more diverse cohorts and standardized input conditions is essential to fully evaluate the reproducibility, consistency, and construct validity of generative AI in medical education. Rather than replacing human evaluators, generative AI could play a complementary role in hybrid evaluation models, supporting fairness, scalability, and feedback generation in OSCE.

## Appendix

Multimedia Appendix 1 English version of the structured prompt.

Multimedia Appendix 2 All ChatGPT conversations used in the preparation of the manuscript.

## Abbreviations

AI: artificial intelligence
ICC: intraclass correlation coefficient
OSCE: objective structured clinical examination
SP: standardized patient

## Data Availability

The datasets generated or analyzed during this study are available from the corresponding author on reasonable request.
