# Supplementary material for: The Validity of Generative Artificial Intelligence in Evaluating Medical Students in Objective Structured Clinical Examination: Experimental Study
Source: JMIR Form Res. 2025 Dec 4;9:e79465. doi: 10.2196/79465 (PMC12715467; doi:10.2196/79465)
Supplement: Multimedia Appendix 1 [file formative_v9i1e79465_app1.docx]

# Appendix: English Translation of the Structured Prompt

Please evaluate the medical interview based on the evaluation criteria (A–C), using a six-point scale (1 = poor, 6 = excellent).

Also, please evaluate the medical record based on the evaluation criteria (D–F), using the same six-point scale.

**********************

Physician: Nice to meet you. Hello.

Patient: Nice to meet you.

Physician: I’m Tanaka, a medical student who will be examining you today. Thank you for your time.

Patient: Thank you.

Physician: May I confirm your full name?

Patient: Yes, my name is Hanako Yamada.

Physician: Hanako Yamada. Thank you. May I also ask your age for confirmation?

Patient: Yes, I’m 48 years old.

Physician: Thank you. What brings you in today?

Patient: Well, I’ve been having a bit of stomach pain.

Physician: Stomach pain, I see. Could you tell me more about the pain?

Patient: Yes, doctor, please feel free to ask anything.

Physician: When did the pain start?

Patient: About two weeks ago.

Physician: Has the location or intensity of the pain changed since it started?

Patient: It started around here and hasn’t really changed.

Physician: So the pain started two weeks ago and has remained in the same area?

Patient: Yes.

Physician: And the intensity of the pain—has it changed?

Patient: I think it’s gotten a bit worse.

Physician: I see. If we rate your worst pain ever as 10 out of 10, how would you rate your pain now?

Patient: When it’s weak, it’s a 0, but when it’s strong, it’s about a 7.

Physician: That sounds quite painful.

Patient: Yes, it is.

Physician: Does anything make the pain better or worse?

Patient: When I eat, the pain goes away or eases.

Physician: I see.

Patient: But about two to three hours after eating, it comes back.

Physician: So the pain is mild or absent during meals, but returns as a 7 about two to three hours afterward?

Patient: Yes.

Physician: And outside of those times?

Patient: Not really any pain.

Physician: So the pain mainly comes after meals.

Patient: That’s right.

Physician: Have you had similar pain before?

Patient: No, this is the first time.

Physician: Thank you. May I ask a bit about your medical history?

Patient: Sure.

Physician: Have you had any major illnesses or abnormal results from health checkups?

Patient: Last year I had a urinary tract infection and took antibiotics.

Patient: Also, my knees have hurt for over 10 years. I’ve been taking painkillers.

Physician: Since when?

Patient: More than 10 years ago.

Patient: I also had two cesarean sections.

Physician: When were those?

Patient: About 20 and 15 years ago.

Physician: Thank you. Any significant illnesses in your family?

Patient: My mother is healthy, but my father died of pancreatic cancer at 55.

Physician: How long ago was that?

Patient: About 15 years ago.

Physician: I see. Do you drink or smoke?

Patient: No, I do neither.

Physician: Understood. Are you currently taking any medications?

Patient: Yes, loxoprofen for my knee pain and some stomach medicine.

Physician: Do you remember the name of the stomach medicine?

Patient: No, I forgot.

Physician: Any allergies?

Patient: None.

Physician: Asthma?

Patient: No.

Physician: What is your current occupation?

Patient: I’m a full-time homemaker.

Physician: Do you find that stressful?

Patient: Well, cleaning and laundry... it’s pretty normal.

Physician: Any particular stress?

Patient: Not really.

Physician: Who do you live with?

Patient: My husband and four children.

Physician: So six people in total?

Patient: Yes.

Physician: Regarding your stomach pain that started two weeks ago—did you eat anything unusual around that time?

Patient: Not really anything strange.

Patient: But I do like pizza and hamburgers.

Physician: I see.

Patient: Greasy foods like that seem to worsen the symptoms.

Physician: So, fatty or high-calorie foods may make it worse?

Patient: Yes, that’s how I feel.

Physician: Have you had any recent infections or similar issues?

Patient: No, not recently.

Physician: I see. That’s all for the interview for now.

Patient: Alright.

Physician: I’d like to perform a physical examination, if that’s alright.

Patient: Okay.

Physician: Could you please lie down over here?

Patient: Sure.

Physician: Please remove your top.

Patient: Okay.

Physician: I’ll place a towel over you.

Patient: Alright.

Physician: I’ll lower your pants a little.

Patient: Go ahead.

Physician: Excuse me. Thank you. I’ll now examine your abdomen.

Patient: Okay.

Physician: I’ll use a stethoscope to listen. Is that okay?

Patient: Yes, go ahead.

Physician: Let me know if the stethoscope feels cold.

Patient: Alright.

Physician: Thank you. Now I’ll lightly tap your abdomen.

Patient: Okay.

Physician: Is there any particular area that hurts right now?

Patient: Not at the moment.

Physician: Let me know if any tapping causes pain.

Patient: Yes.

Physician: Excuse me. Are you okay?

Patient: Yes. Should I loosen my belt, doctor?

Physician: Thank you. I’ll check the lower part again. Thank you. Now I’ll gently palpate your abdomen. Please tell me if anything hurts.

Patient: Okay. I feel a bit of discomfort in the upper area.

Physician: Where exactly?

Patient: Where you just touched.

Physician: Around here?

Patient: Yes, around there.

Physician: How about here?

Patient: That area is fine.

Physician: And here?

Patient: Also fine.

Physician: And here?

Patient: Maybe just a little discomfort.

Physician: How about this spot?

Patient: That’s okay.

Physician: And here?

Patient: Fine.

Physician: Now let me check a bit lower. How about here?

Patient: The lower part is totally fine.

Physician: Here too?

Patient: Yes, all good.

Physician: Thank you. That’s the end of the exam. Please get dressed.

Patient: Thank you.

Physician: We’ll proceed with some tests. Do you have any concerns about your abdominal pain?

Patient: My father had pancreatic cancer, so I’m a bit worried about that.

Physician: You’re concerned you might have the same?

Patient: Yes, I’m a little scared.

Physician: Understood. That will be all for today.

*********************
Medical Record

(History of Present Illness)

Epigastric pain began two weeks ago and has shown a progressive worsening pattern, persisting to the present. The pain peaks 2–3 hours after meals and is rated as 7/10 on the Numerical Rating Scale (NRS). The patient reports that the pain intensifies particularly after consuming pizza or hamburgers. No pain is reported during meals or in other daily activities. This is the first episode; the patient has never experienced similar pain in the past.

(Past Medical History)

Urinary tract infection (last year), treated with antibiotics

Chronic knee pain (for over 10 years to present), managed with loxoprofen

Cesarean section (20 and 15 years ago)

(Family History)

Father: Pancreatic cancer (deceased 5 years ago)

(Social History)

No alcohol consumption or smoking

(Current Medications)

Loxoprofen

Over-the-counter gastrointestinal medication

(Allergies)

None (food, drug, asthma: all negative)

(Occupation)

Full-time homemaker; no stress reported

(Physical Examination)

The patient is alert and oriented. Communication is appropriate.

The patient appears overweight.

Inspection reveals no abnormalities.

Auscultation: normal heart sounds.

Percussion: no abnormalities.

Palpation: the patient reports discomfort in the right lower abdomen.

No board-like rigidity or guarding observed.

(Assessment)

Differential Diagnosis #1: Gastric Ulcer

Supporting history: Epigastric pain worsens after meals; pain intensifies with pizza and hamburger consumption

Supporting physical findings: Discomfort in the right lower abdomen

Differential Diagnosis #2: Duodenal Ulcer

Supporting history: Epigastric pain

Supporting physical findings: Discomfort in the right lower abdomen

Differential Diagnosis #3: Pancreatitis

Supporting history: Epigastric pain; worsened by intake of pizza and hamburgers

(Plan)

Blood tests (including CRP, WBC)

Upper gastrointestinal endoscopy

*********************

Evaluation Criteria

A. Consideration for the Patient and Communication

6 — Can perform independently; fully trusted to manage without supervision

5 — Can perform without direct supervision from a supervisor

4 — Can perform under direct supervision

3 — May fail to establish an appropriate physician–patient relationship

2 — Fails to establish an appropriate physician–patient relationship

1 — Causes significant harm to the patient

B. Medical Interview

6 — Can perform independently; fully trusted to manage without supervision

5 — Can perform without direct supervision from a supervisor

4 — Can perform under direct supervision

3 — Fails to collect sufficient information, potentially compromising medical care

2 — Inadequate information collection, leading to compromised medical care

1 — Collects almost no information; clearly interferes with medical care

C. Physical Examination

6 — Can perform independently; fully trusted to manage without supervision

5 — Can perform without direct supervision from a supervisor

4 — Can perform under direct supervision

3 — May interfere with medical care

2 — Interferes with medical care

1 — Clearly interferes with medical care

D. Medical Record Documentation (History and Physical Findings)

6 — Accurately and systematically documents all relevant information obtained during the interview

5 — Accurately documents all relevant information, but lacks systematic structure

4 — Documents most of the relevant information

3 — Incompletely documents relevant information, potentially compromising medical care

2 — Documents some relevant information, but insufficiently; medical care is compromised

1 — Documents almost no relevant information; clearly interferes with medical care

E. Clinical Reasoning

6 — Provides comprehensive differential diagnoses with sound rationale

5 — Provides reasonable differential diagnoses with appropriate rationale

4 — Provides logical explanation only for the primary diagnosis

3 — Provides only superficial differential diagnoses

2 — Fails to provide appropriate differential diagnoses

1 — Unable to generate any differential diagnoses

F. Management

6 — Develops appropriate and specific plans with sufficient detail and proper prioritization

5 — Develops a few appropriate and specific plans with proper prioritization

4 — Develops appropriate plans, but lacks proper prioritization

3 — Develops appropriate plans, but lacks sufficient specificity

2 — Develops plans that are inappropriate or insufficient

1 — Unable to develop a diagnostic or management plan
